# Patient‐Reported Outcome Measures as Predictive Tools for Disease Control in Chronic Rhinosinusitis With Nasal Polyps: A Prospective Study

**DOI:** 10.1002/clt2.70119

**Published:** 2025-11-04

**Authors:** Chen Zhang, Qianqian Zhang, Jiani Chen, Fuying Cheng, Yizhang Wang, Shirui Xue, Yufei Yang, Wenwen Guo, Juan Liu, Dehui Wang, Li Hu, Xicai Sun, Huan Wang, Quan Liu

**Affiliations:** ^1^ Department of Otorhinolaryngology ENT Institute Eye & ENT Hospital Fudan University Shanghai China; ^2^ High Altitude Rhinology Research Center of Eye & ENT Hospital of Fudan University and People's Hospital of Shigatse City Shigatse China; ^3^ Department of Otolaryngology People's Hospital of Shigatse City Shigatse China

**Keywords:** CRS‐PRO, disease control, FESS, rhinosinusitis, SNOT‐22

## Abstract

**Background:**

Chronic rhinosinusitis with nasal polyps (CRSwNP) significantly impairs the quality of life, and disease control is now considered the primary treatment goal. Although patient‐reported outcome measures (PROMs) such as the 22‐item Sinonasal Outcome Test (SNOT‐22) and CRS‐PRO are widely used, their utility in predicting long‐term postoperative disease control remains limited.

**Methods:**

This prospective follow‐up study aimed to evaluate postoperative recovery and identify the predictors of suboptimal disease control in patients with CRSwNP by integrating preoperative PROMs with objective clinical features. A total of 102 patients with CRSwNP undergoing functional endoscopic sinus surgery (FESS) were enrolled, of whom 89 completed at least 12 months of follow‐up. Preoperative and postoperative PROMs were compared across disease control groups classified based on the European Position Paper on Rhinosinusitis and Nasal Polyps 2020 criteria. Least absolute shrinkage and selection operator regression was applied to select objective clinical predictors, which were then combined with either CRS‐PRO or SNOT‐22 item scores to develop and compare the nine machine learning models. Model performance was assessed using area under the curve (AUC), decision curve analysis, sensitivity, specificity, and other metrics.

**Results:**

Eosinophil and neutrophil counts were identified as key objective predictors of suboptimal disease control after FESS. Among all models, logistic regression incorporating CRS‐PRO scores and selected clinical features achieved the best performance, yielding an AUC of 0.866, accuracy of 83.3%, sensitivity of 72.7%, specificity of 89.5%, and F1‐score of 76.2%. This model demonstrated a strong discriminatory ability and potential utility in individualized clinical decision‐making.

**Conclusion:**

Integrating preoperative CRS‐PRO item scores with selected objective clinical parameters enables the accurate prediction of suboptimal disease control in patients with CRSwNP following FESS. This approach supports personalized risk stratification and postoperative management strategies.

## 1 Introduction

Chronic rhinosinusitis with nasal polyps (CRSwNP) is a heterogeneous chronic inflammatory disease affecting the sinonasal mucosa that significantly impairs patients' quality of life (QOL) and imposes a considerable economic burden. Functional endoscopic sinus surgery (FESS) is typically indicated for patients with CRSwNP when appropriate medical therapy fails [1]. Recently, several biologics targeting type 2 inflammation have been approved, indicating their excellent efficacy for CRSwNP. However, CRSwNP remains a challenging and generally incurable condition [2].

Patient‐reported outcome measures (PROMs) are routinely employed to assess the symptom burden and functional impairment in patients with CRS. Among these, the 22‐item SinoNasal Outcome Test (SNOT‐22) is a well‐validated instrument that encompasses nasal symptoms, sleep quality, ear/facial discomfort, and emotional health domains [3]. More recently, the chronic rhinosinusitis patient‐reported outcome (CRS‐PRO) was developed as a concise, patient‐centered alternative tailored specifically for CRS, and it has demonstrated strong reliability and responsiveness in validation studies [4]. We have also translated the CRS‐PRO into a Chinese version and demonstrated its excellent psychometric properties, suggesting that it is a practical and efficient tool for subjective symptom assessment in clinical practice [5].

With the advent of innovative biological therapies for CRSwNP, treatment goals have been redefined, and the concept of disease control has been proposed to evaluate treatment outcomes and guide clinical decision‐making. Although PROMs, including SNOT‐22 and CRS‐PRO, provide valuable insights into subjective symptoms, a novel task has emerged to combine these measures to assess the treatment outcomes of patients with CRSwNP. In this context, increasing attention has been paid to integrating multidimensional data to develop predictive models that support individualized evaluation of outcomes. Therefore, our study aimed to evaluate the utility of the CRS‐PRO in assessing surgical improvement and its association with disease control status in Chinese patients with CRSwNP. Moreover, we first applied a machine learning methodology to construct a predictive model of disease control in patients with CRSwNP undergoing FESS.

## 2 Methods

### 2.1 Study Population

This prospective cohort study enrolled 102 consecutive patients with CRSwNP at the Department of Otorhinolaryngology between February 2021 and July 2023. All patients underwent with standardized pre‐operative assessments and scheduled post‐operative follow‐up. The diagnosis was based on established clinical guidelines, and all patients underwent FESS after failing medical management [1]. The participants were required to complete a minimum follow‐up period of 12 months. The exclusion criteria were as follows: Isolated antrochoanal polyps, fungal rhinosinusitis, cystic fibrosis, and unilateral nasal polyps. This study was approved by the Ethics Committee of the Eye & ENT Hospital of Fudan University, and written informed consent was obtained from all participants following institutional ethical standards. Data collection and processing.

Clinical data included age, sex, number of clinic revisits, smoking history, prior sinus surgery, history of atopy, asthma, aspirin sensitivity, peripheral blood eosinophil and neutrophil counts and percentages, and computed tomography (CT) findings evaluated using the Lund‐Mackay staging system (Table 1). Additional CT metrics, evaluated by an independent radiologist, included the total Lund–Mackay score, ethmoid‐to‐maxillary sinus ratio, ostiomeatal complex score, anterior drainage score (sum of the maxillary, frontal, and anterior ethmoid sinuses), and posterior drainage score (sum of the posterior ethmoid and sphenoid sinuses) [6].

**TABLE 1:** Objective measures of patients' preoperative characteristics.

Characteristic	Controlled (*n* = 56)	Partly controlled (*n* = 17)	Uncontrolled (*n* = 16)	*p*‐value
Gender: Male (%)	37 (66.7%)	13 (76.4%)	11 (68.7%)	0.72
Age, yr, mean ± SD	38.44 ± 11.02	39.18 ± 11.66	41.69 ± 7.32	0.30
Revisit times, m, median (range)	21 (12–39)	21 (12–36)	18 (12–27)	0.49
Smoker (%)	10 (17.9%)	2 (11.8%)	4 (12.5%)	0.77
Atopy (%)	20(35.7%)	7 (41.2%)	6 (37.5%)	0.88
Asthma (%)	11 (16.1%)	4 (17.7%)	4 (25%)	0.71
Aspirin status (%)	4 (7.1%)	1 (5.8%)	1 (6.1%)	0.98
Previous FESS (%)	12 (21.4%)	5 (29.4%)	5 (31.3%)	0.74
Eosinophil number, mean ± SD (× 10^9^/L)	0.26 ± 0.17	0.36 ± 0.25	0.37 ± 0.25	0.02^*^
Eosinophil percent, mean ± SD (%)	3.99 ± 2.59	5.36 ± 3.77	5.53 ± 3.76	0.04^*^
Neutrophil number, mean ± SD (× 10^9^/L)	3.50 ± 1.13	3.73 ± 1.33	4.22 ± 1.36	0.03^*^
Neutrophil precent, mean ± SD (%)	56.96 ± 7.63	54.35 ± 9.12	59.04 ± 7.56	0.62
Total Lund‐Mackay CT score	15.04 ± 3.69	15.47 ± 3.86	16.31 ± 4.39	0.24
Ethmoid sinus CT score	4.79 ± 1.57	5.00 ± 1.73	5.31 ± 1.78	0.25
Frontal sinus CT score	2.43 ± 1.13	2.65 ± 1.17	3.00 ± 1.37	0.08
Sphenoid sinus CT score	1.79 ± 1.02	1.94 ± 0.97	2.00 ± 1.10	0.41
Maxillary sinus CT score	2.34 ± 0.72	2.41 ± 0.79	2.31 ± 0.60	0.98
Ostiomeatal complex CT score	3.70 ± 0.81	3.47 ± 1.13	3.69 ± 1.01	0.77
Ethmoid/maxillary sinus CT ratio	2.13 ± 0.76	2.27 ± 0.91	2.32 ± 0.65	0.32
Anterior drainage CT score	7.55 ± 2.09	7.93 ± 1.82	7.73 ± 2.34	0.66
Posterior drainage CT score	3.96 ± 1.64	4.14 ± 1.66	4.60 ± 1.99	0.21

Abbreviations: CT, computed tomography; FESS, functional endoscopic sinus surgery.

^*^ *p* < 0.05.

Participants completed the EuroQol 5‐dimensional Questionnaire visual analog scale (EQ‐5D‐VAS), CRS‐PRO, and SNOT‐22 both before and after FESS [5, 7]. The CRS‐PRO includes three sub‐domains: Rhino‐psychological symptoms, facial discomfort symptoms, and cough symptoms. The SNOT‐22 includes five sub‐domains: rhinologic symptoms, extra‐nasal rhinologic symptoms, ear/facial symptoms, psychological dysfunction, and sleep dysfunction. Higher scores indicate a significant disease burden. The principal investigator was blinded to all PROM responses throughout the study. With a median follow‐up of 21 months (range: 12–39 months), the disease control status was assessed at each patient's final visit using the European Position Paper on Rhinosinusitis and Nasal Polyps 2020 (EPOS 2020) criteria: controlled (0 criteria), partly controlled (1–2 criteria), and uncontrolled (≥ 3 criteria). Patients were classified as having suboptimal disease control if they met the EPOS 2020 criteria for either partly controlled or uncontrolled disease.

### 2.2 Feature Selection and Data Preprocessing

Least absolute shrinkage and selection operator (LASSO) regression was applied to identify potential predictors of suboptimally controlled CRSwNP using objective clinical parameters. Through 10‐fold cross‐validation, the optimal *λ* threshold (*λ*.min) was determined to select the final panel of key variables while maximizing model predictive accuracy. The selected features were then combined with the preoperative CRS‐PRO or SNOT‐22 individual item scores to build predictive models. Clinically objective parameters were screened and combined with either the preoperative CRS‐PRO or SNOT‐22 item scores for further analysis.

Using “tidymodels” and “caret” packages in R software, the nine machine learning algorithms were implemented: logistic regression, decision tree, random forest, support vector machine, gradient boosting machine, neural network, LASSO, ridge regression, and k‐nearest neighbors (KNN). The dataset was stratified into training (65%) and testing (35%) subsets to preserve the distribution of the outcomes. To assess clinical utility, model performance was evaluated using receiver operating characteristic (ROC) curve analysis based on the area under the curve (AUC)and decision curve analysis (DCA).

Feature importance was evaluated using the “shapviz” package. SHapley Additive exPlanations (SHAP) values were calculated for the optimized logistic regression model, which incorporated the selected objective parameters and preoperative CRS‐PRO item scores as predictors. The stability of feature importance rankings was assessed using 100 bootstrap iterations of the training set. Force plots were used to visualize the contribution of each variable to the individual predictions. The final model was deployed on the Shinyapps.io platform to enable interactive web‐based use.

### 2.3 Statistical Analysis

Statistical analyses were performed using the R software (version 4.4.0) and Prism (version 23.0). Continuous variables are presented as the mean ± standard deviation (SD). Differences in the mean EQ‐5D‐VAS, CRS‐PRO, and SNOT‐22 scores across the three CRS control groups (controlled, partly controlled, and uncontrolled) were assessed using one‐way analysis of variance, followed by Tukey's honest significant difference test for post hoc comparisons. Pre‐ and postoperative PROMs were compared using the Wilcoxon matched‐pair signed rank test. Statistical significance was set at *p* < 0.05.

## 3 Results

### 3.1 Participant Inclusion and Baseline Characteristics

**FIGURE 1:**
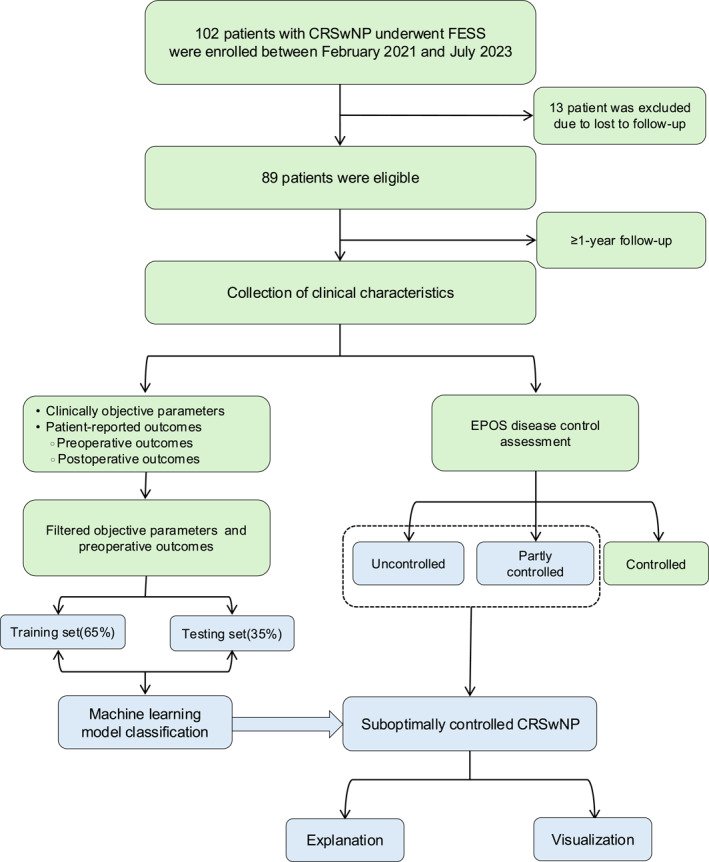
Inclusion flow of this study. CRSwNP, chronic rhinosinusitis with nasal polyp; EPOS, The European Position Paper on Sinusitis; FESS, functional endoscopic sinus surgery.

An overview of the study design and workflow is presented in Figure 1. After excluding 13 patients due to loss to follow‐up, 89 patients completed at least one year of follow‐up and were included in the final analysis. The baseline demographic and clinical characteristics, along with preoperative and postoperative PROMs, are summarized in Table 1. The cohort consisted of 61 males (68.5%) and 28 females, with a mean age of 39.2 years. Among the 89 participants, 16 (17.9%) were current smokers, 46 (51.7%) had a history of atopy, and 19 (21.3%) had comorbid asthma. Six patients (6.7%) reported aspirin sensitivity, and 32 (36%) had a history of prior FESS. The mean peripheral blood eosinophil count was 0.30 × 10^9^/L, while the mean neutrophil count was 3.67 × 10^9^/L. The average Lund‐Mackay CT score for the cohort was 15.35 ± 3.84.

### 3.2 Preoperative and Postoperative PROMs Across Disease Control Groups

PROM comparisons among patients in the controlled (*n* = 56), partly controlled (*n* = 17), and uncontrolled (*n* = 16) CRSwNP groups are presented in Table 2. Compared with patients in the controlled group, those with suboptimally controlled CRSwNP exhibited significantly higher preoperative scores in the CRS‐PRO total score and its rhino‐psychological subdomain, CRS‐PRO total scores and rhino‐psychological subdomain scores, as well as higher SNOT‐22 total scores and rhinologic, ear/facial, psychological, and sleep dysfunction subdomains (*p* < 0.05). These findings suggest that preoperative PROMs may be associated with subsequent disease control.

**TABLE 2:** Preoperative and postoperative patient‐reported outcome measures according to disease control status.

Characteristic	Controlled (*n* = 56)	Partly controlled (*n* = 17)	Uncontrolled (*n* = 16)	*p*‐value (1‐way ANOVA)
CRS‐PRO total				
Preoperative	14.40 ± 5.78	19.35 ± 8.29^a^	19.88 ± 8.19^b^	0.0014*
Postoperative	4.91 ± 3.59	11.94 ± 5.53^a^	19.06 ± 6.67^b^ ^,^ ^c^	< 0.0001*
CRS‐PRO subdomain				
Rhino‐psychological symptoms				
Preoperative	11.95 ± 4.82	15.88 ± 6.19^a^	16.69 ± 7.00^b^	0.0009*
Postoperative	3.64 ± 2.62	9.82 ± 3.97^a^	14.5 ± 5.19^b^ ^,^ ^c^	< 0.0001*
Facial discomfort symptoms				
Preoperative	1.02 ± 1.21	1.47 ± 1.55	1.25 ± 1.44	0.36
Postoperative	0.29 ± 0.62	0.47 ± 0.62	1.38 ± 1.46^b^	< 0.0001*
Cough symptoms				
Preoperative	1.43 ± 1.43	2 ± 1.58	1.94 ± 1.39	0.13
Postoperative	0.98 ± 1.07	1.65 ± 1.73	3.19 ± 2.00^b^ ^,^ ^c^	< 0.0001*
SNOT‐22 total				
Preoperative	26.95 ± 14.94	42.71 ± 20.24^a^	45.38 ± 14.61^b^	< 0.0001*
Postoperative	9.79 ± 7.47	21.18 ± 13.13^a^	46.94 ± 16.74^b^ ^,^ ^c^	< 0.0001*
SNOT‐22 subdomain				
Rhinologic symptoms				
Preoperative	13.16 ± 6.22	16.41 ± 6.01	17.06 ± 6.46^b^	0.01*
Postoperative	4.21 ± 3.40	8.41 ± 4.03^a^	15.19 ± 5.36^b^ ^,^ ^c^	< 0.0001*
Extra nasal rhinologic symptoms				
Preoperative	3.61 ± 3.17	4.53 ± 3.38	4.88 ± 3.32	0.13
Postoperative	1.25 ± 1.70	2.88 ± 2.29^a^	6 ± 3.43^b^ ^,^ ^c^	< 0.0001*
Ear‐facial symptoms				
Preoperative	3.77 ± 3.52	6.77 ± 4.56^a^	7.06 ± 5.26^b^	0.0013*
Postoperative	1.46 ± 1.94	2.65 ± 2.89	7.56 ± 4.63^b^ ^,^ ^c^	< 0.0001*
Psychological dysfunction				
Preoperative	6.75 ± 6.65	12.65 ± 8.14^a^	13.94 ± 6.26^b^	< 0.0001*
Postoperative	3 ± 3.38	6.47 ± 5.63^a^	16.25 ± 7.32^b^ ^,^ ^c^	< 0.0001*
Sleep dysfunction				
Preoperative	5.09 ± 5.49	9.82 ± 6.37^a^	11.06 ± 6.02^b^	< 0.0001*
Postoperative	1.82 ± 2.96	4.65 ± 5.16^a^	10.94 ± 5.01^b^ ^,^ ^c^	< 0.0001*
EQ‐5D‐VAS				
Preoperative	79.02 ± 12.75	64.12 ± 25.31	73.94 ± 17.19	0.069
Postoperative	87.41 ± 8.83	76.41 ± 13.25^a^	69.81 ± 13.70^b^	< 0.0001*

Abbreviations: CRS‐PRO, Chronic Rhinosinusitis Patient‐Reported Outcome; EQ‐5D‐VAS, EuroQol 5‐dimensional Questionnaire visual analog scale; SNOT‐22, 22‐item Sinonasal Outcome Test.

^a^ Significant difference between partly controlled and controlled groups (*p* < 0.05).

^b^ Significant difference between uncontrolled and controlled groups (*p* < 0.05).

^c^ Significant difference between uncontrolled and partly controlled groups (*p* < 0.05).

*Significant difference among the groups using one‐way ANOVA (*p* < 0.05).

**FIGURE 2:**
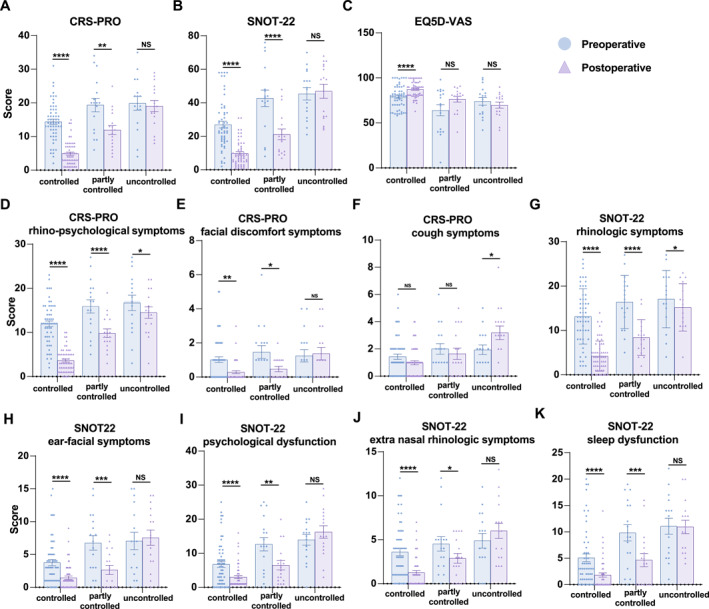
(A–C) Preoperative to post‐operative changes in total CRS‐PRO, SNOT‐22, and EQ‐5D‐VAS scores stratified by disease control status. (D–F) Preoperative to post‐operative changes in CRS‐PRO subdomain scores stratified by disease control status. (G–K) Preoperative to post‐operative changes in the SNOT‐22 subdomain scores stratified by disease control status. CRS‐PRO, Chronic Rhinosinusitis Patient‐Reported Outcome; EQ‐5D‐VAS, EuroQol 5‐dimensional Questionnaire visual analogue scale; SNOT‐22, 22‐item Sinonasal Outcome Test. *; *p* ≤ 0.05; **; *p* ≤ 0.01; ***; *p* ≤ 0.001; ****; *p* ≤ 0.0001.

To assess the impact of FESS, the preoperative and postoperative PROM scores were compared across the three disease control categories (Figure 2). In patients with controlled and partly controlled CRSwNP, statistically significant improvements were observed in the CRS‐PRO total score and its rhino‐psychological and facial discomfort subdomains, and in the SNOT‐22 total score and all five subdomains (rhinologic symptoms, extra‐nasal symptoms, ear/facial symptoms, psychological dysfunction, and sleep dysfunction). However, the cough domain of the CRS‐PRO indicated no postoperative improvement in any group. Moreover, among patients with uncontrolled CRSwNP, improvements were limited to the CRS‐PRO rhino‐psychological and SNOT‐22 rhinologic domains.

Notably, only patients in the controlled group demonstrated a significant postoperative improvement in overall QOL, as measured by EQ‐5D‐VAS scores (*p* < 0.0001). In contrast, patients in the partly controlled group exhibited a statistically non‐significant postoperative improvement in EQ‐5D‐VAS scores (*p* > 0.05), indicating persistent disease burden despite surgery.

### 3.3 The Optimal Prediction Model for Identifying Suboptimally Controlled CRSwNP

**FIGURE 3:**
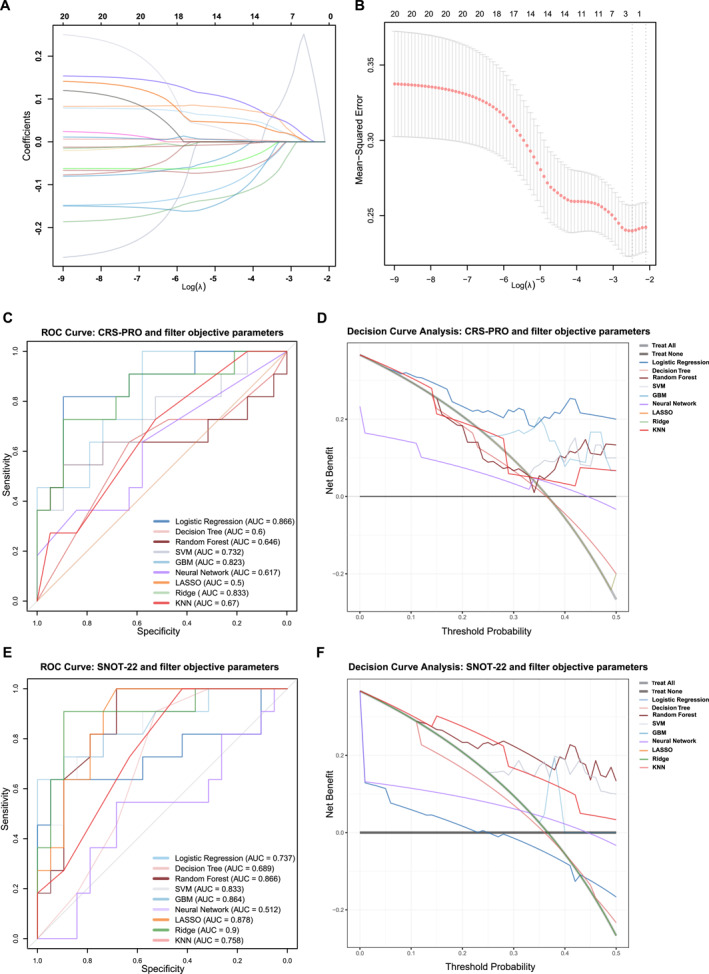
(A) LASSO regression coefficients of clinically objective parameters in the predictive modeling of patients with CRSwNP. (B) LASSO regression with 10‐fold cross‐validation was performed on the clinically objective parameters for feature selection. The vertical dashed lines indicate critical regularization parameters: the left line (*λ*.min) corresponds to the *λ* value yielding the minimum cross‐validation error, whereas the right line (*λ*.1se) represents the largest *λ* value within one standard error of the minimum error, thereby enforcing enhanced model parsimony. (C–D) ROC and DCA were performed based on LASSO‐selected objective parameters and individual CRS‐PRO item scores to evaluate the predictive performance for suboptimally controlled CRSwNP across nine machine learning models. (E–F) ROC and DCA were conducted using LASSO‐selected clinical parameters and individual SNOT‐22 item scores, with predictive performance assessed across the same10 machine learning models. CRS‐PRO, Chronic Rhinosinusitis Patient‐Reported Outcome; CRSwNP, chronic rhinosinusitis with nasal polyp; DCA, Decision Curve Analysis; GBM, gradient boosting machine; KNN, k‐nearest neighbors; LASSO, Least Absolute Shrinkage and Selection Operator; ROC, receiver operating characteristic; SNOT‐22, 22‐item Sinonasal Outcome Test; SVM: support vector machine.

To investigate the association between preoperative characteristics and postoperative disease control, we first applied LASSO regression to a panel of objective clinical parameters, including demographic data, peripheral eosinophil and neutrophil counts and percentages, and Lund‐Mackay CT scores. Using the optimal penalty parameter (*λ*.min), peripheral eosinophil and neutrophil counts emerged as the strongest predictors of poor disease control, with LASSO analysis demonstrating a consistent impact (Figure 3A,B).

We hypothesized that integrating PROMs with objective biomarkers would improve predictive accuracy. Accordingly, the preoperative CRS‐PRO and SNOT‐22 item scores were combined with LASSO‐selected variables to construct multiple predictive models. Following 10‐fold cross‐validation of the training set, the logistic regression model that integrated CRS‐PRO item scores with selected clinical parameters achieved the highest performance on the test set (AUC = 0.866, Figure 3C). The sensitivity, specificity, positive predictive value (PPV), negative predictive value (NPV), F1 score, and accuracy were 83.3%, 72.7%, 89.5%, 80.0%, 85.0%, and 76.2%, respectively (Table 3). DCA further demonstrated consistent net clinical benefit across a broad range of threshold probabilities, underscoring the utility of the model in real‐world decision‐making (Figure 3D).

**TABLE 3:** Performance of each test model using filter clinically objective parameters and individual CRS‐PRO item scores.

Algorithm	AUC	Accuracy	Sensitivity	Specificity	PPV	NPV	F1‐score
Logistic regression	0.866	0.833	0.727	0.895	0.8	0.85	0.762
Decision tree	0.6	0.433	0.727	0.263	0.364	0.625	0.485
Random forest	0.646	0.767	0.455	0.947	0.833	0.75	0.588
SVM	0.732	0.733	0.273	1	1	0.704	0.429
GBM	0.823	0.7	0.182	1	1	0.679	0.308
Neural network	0.617	0.6	0.364	0.737	0.444	0.667	0.4
LASSO	0.5	0.633	0	1	0	0.633	0
Ridge	0.833	0.7	0.182	1	1	0.679	0.308
KNN	0.67	0.7	0.273	0.947	0.75	0.692	0.4

Abbreviations: AUC, area under the curve; GBM, gradient boosting machine; KNN, k‐nearest neighbors; LASSO, Least absolute shrinkage and selection operator; NPV, negative predictive value; PPV, positive predictive value; SVM, support vector machine.

In parallel, models incorporating SNOT‐22 item scores and the same objective variables were developed and evaluated. Ridge regression achieved the highest AUC (0.900), followed by LASSO (0.880) and random forest (0.866) (Figure 3E, Table 4). Although the ridge and LASSO models exhibited strong discriminative performance, the random forest model demonstrated the most balanced trade‐off across multiple metrics, including sensitivity (0.545), specificity (0.895), PPV (0.750), NPV (0.773), and the highest F1 score (0.632). DCA similarly indicated a substantial clinical benefit for the random forest model, supporting its potential practical value in disease control prediction (Figure 3F).

**TABLE 4:** Performance of each test model using filter clinically objective parameters and individual SNOT‐22 item scores.

Algorithm	AUC	Accuracy	Sensitivity	Specificity	PPV	NPV	F1‐score
Logistic regression	0.737	0.467	0.182	0.632	0.222	0.571	0.2
Decision tree	0.689	0.4	0.545	0.316	0.316	0.545	0.4
Random forest	0.866	0.767	0.545	0.895	0.75	0.773	0.632
SVM	0.833	0.733	0.364	0.947	0.8	0.72	0.5
GBM	0.864	0.633	0	1	0	0.633	0
Neural network	0.512	0.6	0.364	0.737	0.444	0.667	0.4
LASSO	0.88	0.733	0.455	0.895	0.714	0.739	0.556
Ridge	0.9	0.633	0	1	0	0.633	0
KNN	0.758	0.667	0.273	0.895	0.6	0.68	0.375

Abbreviations: AUC, area under the curve; GBM, gradient boosting machine; KNN, k‐nearest neighbors; LASSO, Least absolute shrinkage and selection operator; NPV, negative predictive value; PPV, positive predictive value; SVM, support vector machine.

**FIGURE 4:**
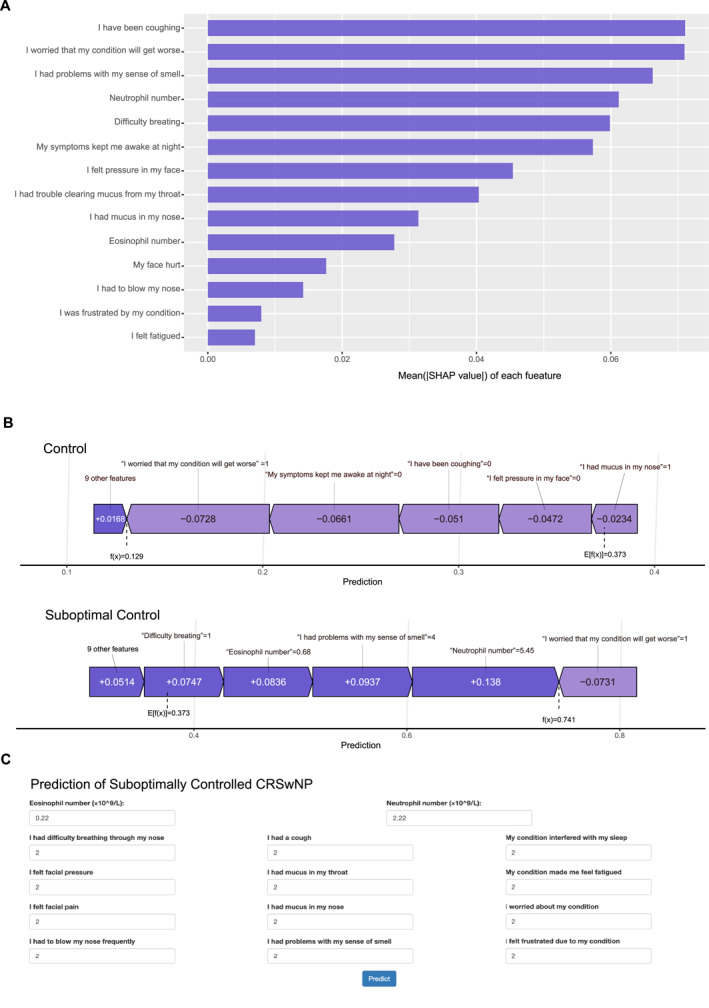
(A) Stability‐assessed feature importance ranking of CRS‐PRO questionnaire items and clinical parameters based on the SHAP value analysis in the logistic regression model. (B) SHAP force plot analysis illustrating individualized prediction interpretation for two representative patient cases, demonstrating the contribution of each CRS‐PRO‐derived feature and clinical variable to the model output. (C) Web‐based interactive risk calculator for suboptimally controlled CRSwNP incorporating the optimized logistic regression model for clinical implementation. CRS‐PRO, Chronic Rhinosinusitis Patient‐Reported Outcome; CRSwNP, chronic rhinosinusitis with nasal polyp; SHAP, Shapley Additive Explanation.

Overall, models based on CRS‐PRO item scores combined with selected clinical features outperformed those based on the SNOT‐22, highlighting the greater predictive value of CRS‐PRO in this framework. To enhance interpretability, the optimal logistic regression model was further analyzed using SHAP. The SHAP‐based feature importance rankings demonstrated stable and consistent contribution patterns across the 100 bootstrap iterations (Figure 4A). The SHAP force plots illustrate the individualized prediction contributions for two representative patients: One with controlled CRSwNP and one with suboptimally controlled CRSwNP (Figure 4B). The directional influence of each feature on the model prediction was visualized, thereby facilitating clinical interpretability. Finally, to enable broader clinical application, the model was deployed as an interactive web‐based tool, accessible at: https://fdeent.shinyapps.io/sccrswnp/(Figure 4C).

## 4 Discussion

Given the chronic disease course of CRSwNP and its refractoriness to current therapies, evaluation of symptom severity and treatment outcomes remains a pivotal issue in both clinical research and practice. The newly developed CRS‐PRO has been translated into various languages and validated as a reliable, concise tool for assessing subjective symptomatology and QOL in patients with CRS [5, 8, 9]. Using disease control as an outcome measure in CRS has gained increasing recognition in recent years, as a systematic review summarized recently [10]. However, few studies have specifically evaluated disease control in patients with CRSwNP. In the current study, we observed significant improvements in EQ‐5D‐VAS, CRS‐PRO, and SNOT‐22 scores among patients with controlled CRSwNP following FESS. Although uncontrolled patients indicated significant improvement in the rhino‐psychological subdomain, no meaningful improvements were observed in the facial discomfort, cough, and sleep subdomains. Moreover, we first applied LASSO regression analysis and constructed machine‐learning models to predict postoperative disease control. Our model, which incorporated CRS‐PRO scores and selected clinical variables demonstrated superior overall performance, supporting the use of individualized postoperative management strategies in patients with CRSwNP.

Lin et al. and our previous report demonstrated the high responsiveness of CRS‐PRO in evaluating the improvement of subjective symptoms after FESS [5, 11]. We extended the follow‐up period and further evaluated the changes in CRS‐PRO and SNOT‐22 scores following FESS in patients with varying disease control statuses. CRS‐PRO and SNOT‐22 indicated significant improvements in symptom scores postoperatively in patients with controlled and partly controlled CRSwNP but not in those with uncontrolled disease. Moreover, consistent with previous reports, patients with higher preoperative CRS‐PRO or SNOT‐22 score are more likely to display suboptimal control status [12]. This may reflect the persistence of residual symptoms in patients with more severe symptoms, even when substantial improvements are achieved after surgery. Collectively, our findings reinforce the value of CRS‐PRO as a useful tool for assessing symptom burden and disease states in patients with CRS [4, 5, 11].

LASSO regression analysis identified peripheral eosinophil and neutrophil counts as key objective predictors of suboptimally controlled CRSwNP. Eosinophilic inflammation has long been recognized as a hallmark of severe CRSwNP, with elevated tissue or peripheral eosinophil levels linked to a higher disease burden, increased risk of polyp recurrence, and poorer surgical outcomes following FESS [13]. Previous studies have revealed that neutrophil infiltration in polyp tissues is associated with disease refractoriness, particularly in Asian CRSwNP populations [14]. Initially, we observed the predictive value of peripheral neutrophils for postoperative disease control in patients with CRSwNP. However, the association between peripheral and polyp‐infiltrated neutrophils remains unknown. Further studies are needed to delineate the phenotype of peripheral neutrophils from CRSwNP and clarify their role in disease persistence and recurrence.

The SHAP analysis revealed that cough, olfactory dysfunction, and difficulty breathing were the strongest patient‐reported contributors to suboptimal disease control. These symptoms may indicate the presence of comorbid asthma or an elevated underlying inflammatory burden. Cough is a common symptom of CRS and a clinical hallmark of asthma, a frequent comorbidity known to exacerbate CRS severity and lead to disease refractoriness [15, 16]. Mucosal inflammation of the upper airway can increase cough reflex sensitivity [17]. Moreover, secretions emanating from the nose and sinuses can stimulate cough‐triggering nerves located in the hypopharynx or larynx [18]. Olfactory dysfunction and breathing difficulty are among the most severe and commonly reported symptoms in patients with CRSwNP [19]. Loss of smell has been established as an indicator of disease burden and a prognostic marker for outcomes following FESS [20]. Similarly, preoperative complaints of breathing difficulty likely reflect a significant sinonasal obstruction caused by polyp volume or mucosal edema. This complaint may serve as a surrogate marker of baseline disease severity, and if not fully addressed by surgery, may contribute to poor postoperative disease control. Our findings suggest that a quantitative evaluation of individual symptoms may benefit preoperative patient counseling, particularly regarding the severity of cough, olfactory function, and breathing difficulties.

Several studies have demonstrated that preoperative symptom scores, such as the SNOT‐22, are strong predictors of outcomes after FESS [21, 22]. In our study, the logistic regression model incorporating CRS‐PRO items and objective clinical parameters achieved an AUC of 0.866, comparable to the models using SNOT‐22 items with objective parameters. These findings indicate that both PROMs can be used effectively to predict postoperative disease control. Importantly, CRS‐PRO's concise structure minimizes patient burden while retaining strong psychometric validity, making it a particularly practical tool for clinical use. Furthermore, our findings align with the 2023 EPOS/EUFOREA guidelines [23], which formally recognize a SNOT‐22 score ≥ 40 as a key criterion for identifying significantly impaired QOL and eligibility for biological therapy. This concordance highlights the clinical relevance of our model and supports the utility of PROMs, such as CRS‐PRO, in informed treatment decision‐making.

This study has several limitations. The relatively small sample size from a single center may limit the statistical power and increase the risk of overfitting in machine‐learning modeling. Nonetheless, we employed several measures to enhance model robustness, including rigorous internal validation strategies, such as k‐fold cross‐validation and LASSO regularization, which help mitigate overfitting and improve the stability of feature selection. Despite these efforts, the model has not yet been externally validated in an independent cohort from a different institution or geographical region. Therefore, the generalizability of internally validated performance metrics to broader populations remains to be confirmed, and the clinical applicability of the predictive tool requires further verification.

As one of the initial attempts to integrate patient‐reported outcomes and clinical parameters for predicting postoperative control in CRSwNP using machine learning, this study provides a foundational framework and valuable preliminary insights. We believe that our approach represents a significant step forward in this field and may help inform the design of future larger‐scale multicenter studies. Subsequent research should focus on expanding the sample size, incorporating external validation, and exploring additional clinical, imaging, or molecular biomarkers to enhance the robustness and translational potential of the model.

In conclusion, CRS‐PRO and SNOT‐22 are promising tools for assessing postoperative symptoms and predicting suboptimal disease control in patients with CRSwNP following FESS. In our cohort, the CRS‐PRO demonstrated responsiveness comparable to that of the SNOT‐22 across varying levels of disease control, and both instruments indicated similar predictive accuracy. While these results are encouraging, the limitations related to the sample size and lack of external validation necessitate cautious interpretation. The proposed model holds potential as a future clinical decision‐support tool; however, its real‐world applicability and generalizability require further confirmation through larger prospective multicenter validation studies.

## Author Contributions

**Chen Zhang:** writing – original draft, software, formal analysis, writing – review and editing, conceptualization, methodology, visualization, data curation. **Qianqian Zhang:** data curation, software, methodology, visualization, investigation, conceptualization. **Jiani Chen:** formal analysis, investigation, validation, conceptualization. **Fuying Cheng:** validation, investigation. **Yizhang Wang:** methodology, formal analysis. **Shirui Xue:** methodology. **Yufei Yang:** methodology. **Wenwen Guo:** methodology. **Juan Liu:** methodology. **Dehui Wang:** project administration. **Li Hu:** writing – review and editing, project administration, resources. **Xicai Sun:** supervision, project administration, funding acquisition, writing – review and editing. **Huan Wang:** supervision, writing – review and editing, funding acquisition, resources, project administration, writing – original draft. **Quan Liu:** supervision, project administration, writing – review and editing, resources, funding acquisition.

## Conflicts of Interest

The authors declare no conflicts of interest.

## Data Availability

The data that support the findings of this study are available on request from the corresponding author. The data are not publicly available due to privacy or ethical restrictions.
